# *catena*-Poly[(triaqua­cadmium)-μ-5-hy­droxy­isophthalato-κ^3^ *O* ^1^,*O* ^1′^:*O* ^3^]

**DOI:** 10.1107/S160053681104236X

**Published:** 2011-10-22

**Authors:** Xiao-Hong Wei, Jun Zhao

**Affiliations:** a College of Mechanical & Material Engineering, ChinaThree Gorges University, Yichang 443002, People’s Republic of China

## Abstract

The title compound, [Cd(C_8_H_4_O_5_)(H_2_O)_3_]_*n*_, a one-dimensional chain complex of 5-hy­droxy­isophthalate with Cd^II^, was prepared by a hydro­thermal reaction. The Cd^II^ ion is coordinated by three water O atoms and three carboxyl­ate O atoms of two different 5-hy­droxy­isophthalate ligands, which act as bidentate and monodentate ligands. The crystal structure is stabilized by O—H⋯O hydrogen bonds.

## Related literature

For applications of coordination polymers in functional materials, see: Inoue *et al.* (2001 ▶). For coordination polymers including benzene­dicarboxyl­ates and their derivatives, see: Xiao *et al.* (2004 ▶); Plater *et al.* (2001 ▶); Zhao *et al.* (2011 ▶).
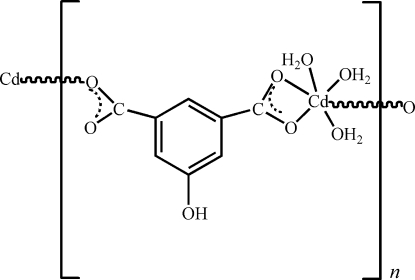


## Experimental

### 

#### Crystal data

[Cd(C_8_H_4_O_5_)(H_2_O)_3_]
*M* *_r_* = 346.56
Orthorhombic, 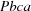

*a* = 8.027 (3) Å
*b* = 13.582 (5) Å
*c* = 19.591 (7) Å
*V* = 2135.7 (14) Å^3^
*Z* = 8
Mo *K*α radiation
μ = 2.08 mm^−1^
*T* = 296 K
0.23 × 0.20 × 0.18 mm

#### Data collection

Bruker SMART CCD diffractometer
Absorption correction: multi-scan (*SADABS*; Sheldrick, 1996 ▶) *T*_min_ = 0.647, *T*_max_ = 0.702
17155 measured reflections
1874 independent reflections
1781 reflections with *I* > 2σ(*I*)
*R*_int_ = 0.074

#### Refinement

*R*[*F* ^2^ > 2σ(*F* ^2^)] = 0.023
*wR*(*F* ^2^) = 0.063
*S* = 1.00
1874 reflections
176 parameters
10 restraints
H atoms treated by a mixture of independent and constrained refinement
Δρ_max_ = 0.64 e Å^−3^
Δρ_min_ = −0.48 e Å^−3^

### 

Data collection: *SMART* (Bruker, 1999 ▶); cell refinement: *SAINT* (Bruker, 1999 ▶); data reduction: *SAINT*; program(s) used to solve structure: *SHELXS97* (Sheldrick, 2008 ▶); program(s) used to refine structure: *SHELXL97* (Sheldrick, 2008 ▶); molecular graphics: *SHELXTL* (Sheldrick, 2008 ▶); software used to prepare material for publication: *SHELXTL*.

## Supplementary Material

Crystal structure: contains datablock(s) I, global. DOI: 10.1107/S160053681104236X/bt5673sup1.cif

Structure factors: contains datablock(s) I. DOI: 10.1107/S160053681104236X/bt5673Isup2.hkl

Additional supplementary materials:  crystallographic information; 3D view; checkCIF report

## Figures and Tables

**Table 1:** Hydrogen-bond geometry (Å, °)

*D*—H⋯*A*	*D*—H	H⋯*A*	*D*⋯*A*	*D*—H⋯*A*
O5—H5*A*⋯O2^i^	0.86 (1)	1.79 (1)	2.645 (2)	178 (3)
O8—H8*A*⋯O1^ii^	0.86 (1)	1.89 (2)	2.719 (3)	161 (3)
O7—H7*B*⋯O3^iii^	0.86 (1)	1.98 (2)	2.790 (3)	156 (3)
O6—H6*A*⋯O5^ii^	0.86 (1)	1.89 (1)	2.748 (3)	178 (3)
O7—H7*A*⋯O8^iv^	0.86 (1)	2.18 (2)	2.947 (3)	149 (3)
O8—H8*B*⋯O4^v^	0.86 (1)	2.08 (2)	2.869 (3)	154 (3)
O6—H6*B*⋯O3^vi^	0.86 (1)	1.94 (1)	2.781 (3)	168 (3)

Symmetry codes: (i) 
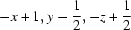
; (ii) 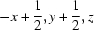; (iii) 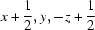; (iv) 
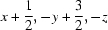
; (v) 
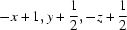
; (vi) 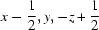.

**Fig. 1.**
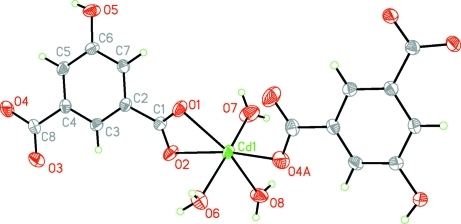
An ORTEP representation of the structure of the title compound showing the atomic numbering and 50% probability displacement ellipsoids [symmetry code: A: -x + 1/2,-y + 1,z - 1/2].

**Fig. 2.**
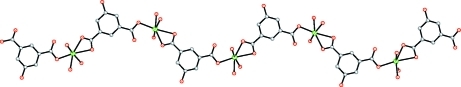
A portion of polymeric zigzag chain in the title compound. H atoms are omitted for clarity.

## supplementary crystallographic information

### Comment

The rational design and construction of coordination polymers has attracted much attention owing to their intriguing topologies and potential applications as functional materials (Inoue *et al.*, 2001). Benzenedicarboxylates and their derivatives have been extensively employed to link metal ions in the synthesis of one-, two- or three-dimensional structures. They often act as bridging or chelating ligands (Xiao *et al.*, 2004; Plater *et al.*, 2001). In continuation of our study of the chemistry of benzenedicarboxylate ligands (Zhao *et al.*, 2011), we present here the title compound, in which the 5- hydroxyisophthalate dianion functions as a bridge between adjacent Cd^II^ centers. In the title compound, [Cd(C_8_H_4_O_5_)(H_2_O)_3_]*_n_*, Cd^II^ ion is hexacoordinated in a distorted octahedral geometry by three O atoms from two organic ligands and three water molecules (Fig. 1). Each ligand  bridges two Cd^II^ ions, that results in formation of polymeric zigzag chains extended along the direction [001] (Fig. 2). The crystal packing is stabilized by extensive O–H···O hydrogen bonds (Table 1).

### Experimental

A mixture of 5-hydroxyisophthalic acid (0.0182 g, 0.1 mmol), Cd(CH_3_COO)_2_.2H_2_O (0.0266 g, 0.1 mmol), water (8 mL) was stired vigorously for 30 min and then sealed in a Teflon-lined stainless-steel autoclave. The autoclave was heated and maintained at 413 K for 3days, and then cooled to room temperature at 5 K h^-1^ to obtain colorless prism crystals suitable for X-ray analysis.

### Refinement

All H-atoms bonded to C were positioned geometrically and refined using a riding model with C–H = 0.93 Å, *U*_iso_(H) = 1.2 *U*_eq_(C) for aromatic hydrogen atoms. The H-atoms bonded to  O were located in a difference Fourier map and their coordinates were refined. The O-H distance was restrained to with O–H = 0.86 (1)Å and the H···H distances in the water molecules to 1.39 (1)Å. *U*_iso_(H) was set to 1.5 *U*_eq_(O).

### Crystal data

**Table:**

[Cd(C_8_H_4_O_5_)(H_2_O)_3_]	4
*M**_r_* = 346.56	*D*_x_ = 2.156 Mg m^−^^3^
Orthorhombic, *P**b**c**a*	Mo *K*α radiation, λ = 0.71073 Å
Hall symbol: -P 2ac 2ab	Cell parameters from 5687 reflections
*a* = 8.027 (3) Å	θ = 3.1–25.0°
*b* = 13.582 (5) Å	µ = 2.08 mm^−^^1^
*c* = 19.591 (7) Å	*T* = 296 K
*V* = 2135.7 (14) Å^3^	Prism, colourless
*Z* = 8	0.23 × 0.20 × 0.18 mm
*F*(000) = 1360	

### Data collection

**Table:**

Bruker SMART CCD diffractometer	1874 independent reflections
Radiation source: fine-focus sealed tube	1781 reflections with *I* > 2σ(*I*)
graphite	*R*_int_ = 0.074
phi and ω scans	θ_max_ = 25.0°, θ_min_ = 3.1°
Absorption correction: multi-scan (*SADABS*; Sheldrick, 1996)	*h* = −9→9
*T*_min_ = 0.647, *T*_max_ = 0.702	*k* = −16→16
17155 measured reflections	*l* = −23→23

### Refinement

**Table:**

Refinement on *F*^2^	Secondary atom site location: difference Fourier map
Least-squares matrix: full	Hydrogen site location: inferred from neighbouring sites
*R*[*F*^2^ > 2σ(*F*^2^)] = 0.023	H atoms treated by a mixture of independent and constrained refinement
*wR*(*F*^2^) = 0.063	*w* = 1/[σ^2^(*F*_o_^2^) + (0.030*P*)^2^ + 2.147*P*] where *P* = (*F*_o_^2^ + 2*F*_c_^2^)/3
*S* = 1.00	(Δ/σ)_max_ = 0.002
1874 reflections	Δρ_max_ = 0.64 e Å^−^^3^
176 parameters	Δρ_min_ = −0.48 e Å^−^^3^
10 restraints	Extinction correction: *SHELXL97* (Sheldrick, 2008), Fc^*^=kFc[1+0.001xFc^2^λ^3^/sin(2θ)]^-1/4^
Primary atom site location: structure-invariant direct methods	Extinction coefficient: 0.0042 (2)

### Special details

**Table:**

Geometry. All e.s.d.'s (except the e.s.d. in the dihedral angle between two l.s. planes) are estimated using the full covariance matrix. The cell e.s.d.'s are taken into account individually in the estimation of e.s.d.'s in distances, angles and torsion angles; correlations between e.s.d.'s in cell parameters are only used when they are defined by crystal symmetry. An approximate (isotropic) treatment of cell e.s.d.'s is used for estimating e.s.d.'s involving l.s. planes.
Refinement. Refinement of *F*^2^ against ALL reflections. The weighted *R*-factor *wR* and goodness of fit *S* are based on *F*^2^, conventional *R*-factors *R* are based on *F*, with *F* set to zero for negative *F*^2^. The threshold expression of *F*^2^ > σ(*F*^2^) is used only for calculating *R*-factors(gt) *etc*. and is not relevant to the choice of reflections for refinement. *R*-factors based on *F*^2^ are statistically about twice as large as those based on *F*, and *R*- factors based on ALL data will be even larger.

### Fractional atomic coordinates and isotropic or equivalent isotropic displacement parameters (Å^2^)

**Table:**

	*x*	*y*	*z*	*U*_iso_*/*U*_eq_	
Cd1	0.24003 (2)	0.627985 (11)	0.051993 (8)	0.02196 (12)	
O1	0.2744 (2)	0.47757 (12)	0.10249 (8)	0.0306 (4)	
O2	0.3061 (2)	0.60170 (12)	0.17270 (8)	0.0291 (4)	
O3	0.2644 (2)	0.48696 (15)	0.42628 (10)	0.0439 (5)	
O4	0.3590 (3)	0.33884 (15)	0.44912 (8)	0.0398 (5)	
O5	0.5561 (2)	0.19980 (12)	0.22523 (8)	0.0363 (4)	
H5A	0.601 (4)	0.169 (2)	0.2588 (11)	0.054*	
O6	−0.0295 (2)	0.64996 (15)	0.08972 (9)	0.0364 (4)	
H6A	−0.039 (4)	0.664 (2)	0.1324 (6)	0.055*	
H6B	−0.100 (3)	0.6046 (18)	0.0802 (14)	0.055*	
O7	0.5220 (3)	0.62281 (14)	0.03440 (14)	0.0509 (6)	
H7B	0.579 (4)	0.5698 (14)	0.0398 (18)	0.076*	
H7A	0.587 (3)	0.6668 (17)	0.0178 (18)	0.076*	
O8	0.2910 (3)	0.79576 (13)	0.05101 (9)	0.0365 (4)	
H8A	0.257 (3)	0.8459 (18)	0.0741 (17)	0.055*	
H8B	0.3899 (19)	0.808 (2)	0.0371 (15)	0.055*	
C1	0.3094 (3)	0.51031 (15)	0.16111 (10)	0.0222 (5)	
C2	0.3560 (3)	0.43938 (15)	0.21601 (11)	0.0212 (4)	
C3	0.3239 (3)	0.45950 (16)	0.28430 (11)	0.0229 (5)	
H3	0.2752	0.5189	0.2970	0.027*	
C4	0.3659 (3)	0.38923 (15)	0.33382 (11)	0.0222 (5)	
C5	0.4416 (3)	0.30120 (16)	0.31452 (11)	0.0250 (5)	
H5	0.4676	0.2541	0.3473	0.030*	
C6	0.4780 (3)	0.28385 (17)	0.24653 (11)	0.0243 (5)	
C7	0.4336 (3)	0.35181 (17)	0.19718 (11)	0.0235 (5)	
H7	0.4555	0.3390	0.1514	0.028*	
C8	0.3255 (3)	0.40683 (18)	0.40740 (11)	0.0259 (5)	

### Atomic displacement parameters (Å^2^)

**Table:**

	*U*^11^	*U*^22^	*U*^33^	*U*^12^	*U*^13^	*U*^23^
Cd1	0.02666 (16)	0.02087 (17)	0.01835 (16)	0.00125 (6)	−0.00444 (6)	0.00390 (5)
O1	0.0507 (10)	0.0204 (8)	0.0207 (8)	−0.0003 (7)	−0.0092 (7)	0.0013 (6)
O2	0.0432 (10)	0.0181 (7)	0.0261 (8)	−0.0005 (8)	−0.0100 (8)	0.0009 (7)
O3	0.0584 (13)	0.0363 (11)	0.0370 (10)	0.0048 (9)	0.0181 (9)	−0.0067 (9)
O4	0.0525 (12)	0.0485 (11)	0.0185 (8)	0.0138 (10)	0.0072 (7)	0.0065 (7)
O5	0.0566 (12)	0.0295 (9)	0.0228 (8)	0.0199 (8)	−0.0079 (8)	−0.0036 (7)
O6	0.0338 (10)	0.0444 (10)	0.0309 (9)	−0.0040 (8)	0.0052 (8)	−0.0034 (8)
O7	0.0292 (11)	0.0421 (13)	0.0815 (16)	0.0072 (8)	0.0138 (11)	0.0168 (10)
O8	0.0405 (10)	0.0221 (9)	0.0469 (11)	−0.0029 (8)	0.0122 (8)	−0.0071 (7)
C1	0.0245 (11)	0.0203 (11)	0.0218 (11)	−0.0009 (9)	−0.0020 (9)	0.0010 (8)
C2	0.0227 (11)	0.0202 (10)	0.0207 (10)	−0.0024 (9)	−0.0023 (8)	0.0018 (8)
C3	0.0255 (11)	0.0201 (11)	0.0229 (10)	0.0004 (9)	−0.0002 (9)	0.0003 (8)
C4	0.0239 (11)	0.0243 (11)	0.0184 (11)	−0.0032 (9)	−0.0007 (9)	0.0013 (8)
C5	0.0309 (12)	0.0229 (11)	0.0212 (11)	0.0012 (9)	−0.0038 (9)	0.0047 (9)
C6	0.0284 (11)	0.0204 (11)	0.0241 (11)	0.0035 (9)	−0.0038 (9)	−0.0007 (8)
C7	0.0291 (12)	0.0250 (10)	0.0164 (10)	0.0013 (10)	−0.0012 (9)	−0.0004 (8)
C8	0.0235 (11)	0.0336 (12)	0.0207 (11)	−0.0043 (10)	0.0026 (9)	−0.0006 (10)

### Geometric parameters (Å, °)

**Table:**

Cd1—O4^i^	2.2129 (17)	O7—H7B	0.860 (10)
Cd1—O1	2.2865 (17)	O7—H7A	0.856 (10)
Cd1—O7	2.290 (2)	O8—H8A	0.862 (10)
Cd1—O6	2.306 (2)	O8—H8B	0.857 (10)
Cd1—O8	2.315 (2)	C1—C2	1.492 (3)
Cd1—O2	2.4496 (18)	C2—C3	1.389 (3)
Cd1—C1	2.726 (2)	C2—C7	1.392 (3)
O1—C1	1.263 (3)	C3—C4	1.402 (3)
O2—C1	1.262 (3)	C3—H3	0.9300
O3—C8	1.250 (3)	C4—C5	1.394 (3)
O4—C8	1.262 (3)	C4—C8	1.497 (3)
O4—Cd1^ii^	2.2129 (17)	C5—C6	1.384 (3)
O5—C6	1.368 (3)	C5—H5	0.9300
O5—H5A	0.857 (10)	C6—C7	1.383 (3)
O6—H6A	0.861 (10)	C7—H7	0.9300
O6—H6B	0.855 (10)		
			
O4^i^—Cd1—O1	128.26 (7)	Cd1—O8—H8A	136 (2)
O4^i^—Cd1—O7	102.96 (9)	Cd1—O8—H8B	111.2 (19)
O1—Cd1—O7	85.33 (7)	H8A—O8—H8B	107.6 (15)
O4^i^—Cd1—O6	85.89 (7)	O2—C1—O1	120.34 (19)
O1—Cd1—O6	95.16 (7)	O2—C1—C2	120.71 (19)
O7—Cd1—O6	168.36 (8)	O1—C1—C2	118.95 (19)
O4^i^—Cd1—O8	81.69 (7)	O2—C1—Cd1	63.92 (11)
O1—Cd1—O8	149.51 (7)	O1—C1—Cd1	56.50 (11)
O7—Cd1—O8	81.63 (7)	C2—C1—Cd1	174.35 (15)
O6—Cd1—O8	92.35 (7)	C3—C2—C7	120.40 (19)
O4^i^—Cd1—O2	170.62 (7)	C3—C2—C1	121.38 (19)
O1—Cd1—O2	54.97 (5)	C7—C2—C1	118.22 (19)
O7—Cd1—O2	85.81 (8)	C2—C3—C4	119.2 (2)
O6—Cd1—O2	84.98 (7)	C2—C3—H3	120.4
O8—Cd1—O2	96.50 (6)	C4—C3—H3	120.4
O4^i^—Cd1—C1	155.06 (7)	C5—C4—C3	120.1 (2)
O1—Cd1—C1	27.43 (6)	C5—C4—C8	119.52 (19)
O7—Cd1—C1	84.16 (8)	C3—C4—C8	120.4 (2)
O6—Cd1—C1	90.92 (7)	C6—C5—C4	119.95 (19)
O8—Cd1—C1	123.19 (7)	C6—C5—H5	120.0
O2—Cd1—C1	27.56 (6)	C4—C5—H5	120.0
C1—O1—Cd1	96.07 (13)	O5—C6—C7	117.51 (19)
C1—O2—Cd1	88.52 (12)	O5—C6—C5	122.17 (19)
C8—O4—Cd1^ii^	111.35 (15)	C7—C6—C5	120.3 (2)
C6—O5—H5A	111 (2)	C6—C7—C2	120.01 (19)
Cd1—O6—H6A	115 (2)	C6—C7—H7	120.0
Cd1—O6—H6B	117 (2)	C2—C7—H7	120.0
H6A—O6—H6B	108.3 (16)	O3—C8—O4	122.0 (2)
Cd1—O7—H7B	122 (2)	O3—C8—C4	120.6 (2)
Cd1—O7—H7A	130 (2)	O4—C8—C4	117.4 (2)
H7B—O7—H7A	107.9 (16)		
			
O4^i^—Cd1—O1—C1	−170.85 (13)	O6—Cd1—C1—C2	136.9 (16)
O7—Cd1—O1—C1	86.31 (15)	O8—Cd1—C1—C2	−129.7 (16)
O6—Cd1—O1—C1	−82.01 (14)	O2—Cd1—C1—C2	−145.8 (17)
O8—Cd1—O1—C1	21.6 (2)	O2—C1—C2—C3	29.9 (3)
O2—Cd1—O1—C1	−1.84 (13)	O1—C1—C2—C3	−150.7 (2)
O4^i^—Cd1—O2—C1	115.2 (4)	Cd1—C1—C2—C3	173.9 (15)
O1—Cd1—O2—C1	1.83 (13)	O2—C1—C2—C7	−150.0 (2)
O7—Cd1—O2—C1	−85.41 (14)	O1—C1—C2—C7	29.5 (3)
O6—Cd1—O2—C1	101.73 (14)	Cd1—C1—C2—C7	−5.9 (17)
O8—Cd1—O2—C1	−166.47 (14)	C7—C2—C3—C4	−2.0 (3)
Cd1—O2—C1—O1	−3.1 (2)	C1—C2—C3—C4	178.2 (2)
Cd1—O2—C1—C2	176.31 (19)	C2—C3—C4—C5	1.2 (3)
Cd1—O1—C1—O2	3.4 (2)	C2—C3—C4—C8	−177.1 (2)
Cd1—O1—C1—C2	−176.08 (17)	C3—C4—C5—C6	1.1 (3)
O4^i^—Cd1—C1—O2	−159.52 (16)	C8—C4—C5—C6	179.4 (2)
O1—Cd1—C1—O2	−176.8 (2)	C4—C5—C6—O5	178.2 (2)
O7—Cd1—C1—O2	92.13 (15)	C4—C5—C6—C7	−2.6 (3)
O6—Cd1—C1—O2	−77.29 (14)	O5—C6—C7—C2	−179.0 (2)
O8—Cd1—C1—O2	16.12 (17)	C5—C6—C7—C2	1.8 (3)
O4^i^—Cd1—C1—O1	17.2 (2)	C3—C2—C7—C6	0.5 (3)
O7—Cd1—C1—O1	−91.12 (15)	C1—C2—C7—C6	−179.6 (2)
O6—Cd1—C1—O1	99.46 (14)	Cd1^ii^—O4—C8—O3	9.0 (3)
O8—Cd1—C1—O1	−167.13 (13)	Cd1^ii^—O4—C8—C4	−172.58 (16)
O2—Cd1—C1—O1	176.8 (2)	C5—C4—C8—O3	176.8 (2)
O4^i^—Cd1—C1—C2	54.7 (16)	C3—C4—C8—O3	−4.9 (3)
O1—Cd1—C1—C2	37.4 (15)	C5—C4—C8—O4	−1.6 (3)
O7—Cd1—C1—C2	−53.7 (16)	C3—C4—C8—O4	176.7 (2)

Symmetry codes: (i) −*x*+1/2, −*y*+1, *z*−1/2; (ii) −*x*+1/2, −*y*+1, *z*+1/2.

### Hydrogen-bond geometry (Å, °)

**Table:**

*D*—H···*A*	*D*—H	H···*A*	*D*···*A*	*D*—H···*A*
O5—H5A···O2^iii^	0.86 (1)	1.79 (1)	2.645 (2)	178 (3)
O8—H8A···O1^iv^	0.86 (1)	1.89 (2)	2.719 (3)	161 (3)
O7—H7B···O3^v^	0.86 (1)	1.98 (2)	2.790 (3)	156 (3)
O6—H6A···O5^iv^	0.86 (1)	1.89 (1)	2.748 (3)	178 (3)
O7—H7A···O8^vi^	0.86 (1)	2.18 (2)	2.947 (3)	149 (3)
O8—H8B···O4^vii^	0.86 (1)	2.08 (2)	2.869 (3)	154 (3)
O6—H6B···O3^viii^	0.86 (1)	1.94 (1)	2.781 (3)	168 (3)

Symmetry codes: (iii) −*x*+1, *y*−1/2, −*z*+1/2; (iv) −*x*+1/2, *y*+1/2, *z*; (v) *x*+1/2, *y*, −*z*+1/2; (vi) *x*+1/2, −*y*+3/2, −*z*; (vii) −*x*+1, *y*+1/2, −*z*+1/2; (viii) *x*−1/2, *y*, −*z*+1/2.
